# Endovascular therapy for severely calcified plaque at the superficial femoral artery using myocardial biopsy forceps

**DOI:** 10.1186/s42155-021-00257-z

**Published:** 2021-09-15

**Authors:** Shojiro Hirano, Atsushi Funatsu, Shigeru Nakamura, Takanori Ikeda

**Affiliations:** 1 Division of Cardiovascular Medicine, Department of Internal Medicine, Toho University, 5-21-16 Omorinishi, Ota-ku 143-8540 Tokyo, Japan; 2 Cardiovascular Center, Kyoto Katsura Hospital, Kyoto, Japan; 3 Department of Cardiovascular Medicine, Toho University Faculty of Medicine Graduate School of Medicine, Tokyo, Ota-ku Japan

**Keywords:** Endovascular therapy, Superficial femoral artery, Chronic total occlusions

## Abstract

**Background:**

Currently, the success rate of EVT for treating CTO of the SFA is high; however, EVT is still found to be insufficient in treating CTOs with severely calcified lesions. Even if the guidewire crosses the lesion, the calcifications may still cause difficulties during stent expansion.

**Main text:**

A 78-year-old male had been reported to have intermittent claudication with chronic total occlusion (CTO) of the right superficial femoral artery (SFA). Angiography revealed severely calcified plaque (Angiographic calcium score: Group4a [1]) at the ostium of the SFA. Stenting posed a risk of underexpansion, causing the plaque to shift to the deep femoral artery. we decided to remove the calcified plaque using biopsy forceps. After removing the extended calcified plaque, the guidewire could cross easily, and the self-expandable stent was well dilated without causing the plaque to shift to the DFA.

**Conclusions:**

Biopsy forceps may be used in some endovascular cases to remove severely calcified lesions.

To ensure the safety of the patient, the physician must be adept at performing this technique before attempting it.

## Introduction

Currently, the technical success rate of endovascular therapy (EVT) for treating chronic total occlusion (CTO) of the superficial femoral artery (SFA) has been recorded to be over 95 % [1]. The use of EVT for treating above-the-knee vascular lesions has become popular because its currently used devices and techniques are well-developed [3, 4]. However, in treating CTOs with severely calcified lesions, EVT is still found to be insufficient. Even if the guidewire crosses the lesion, the calcifications may still cause difficulties during stent expansion.

In recent years, several EVT techniques for removing calcified lesions, such as the crossbow technique (Crosser® supported by a bent 0.014-in wire) [5] and Pierce technique (percutaneous direct needle puncture of the calcified plaque) [6], have been reported. However, these techniques have shown a limited ability in reducing calcium volume.

Herein, we report a successful case of EVT using biopsy forceps in removing a CTO with severe calcification at the ostium of the SFA.

## Main text

**Fig. 1:**
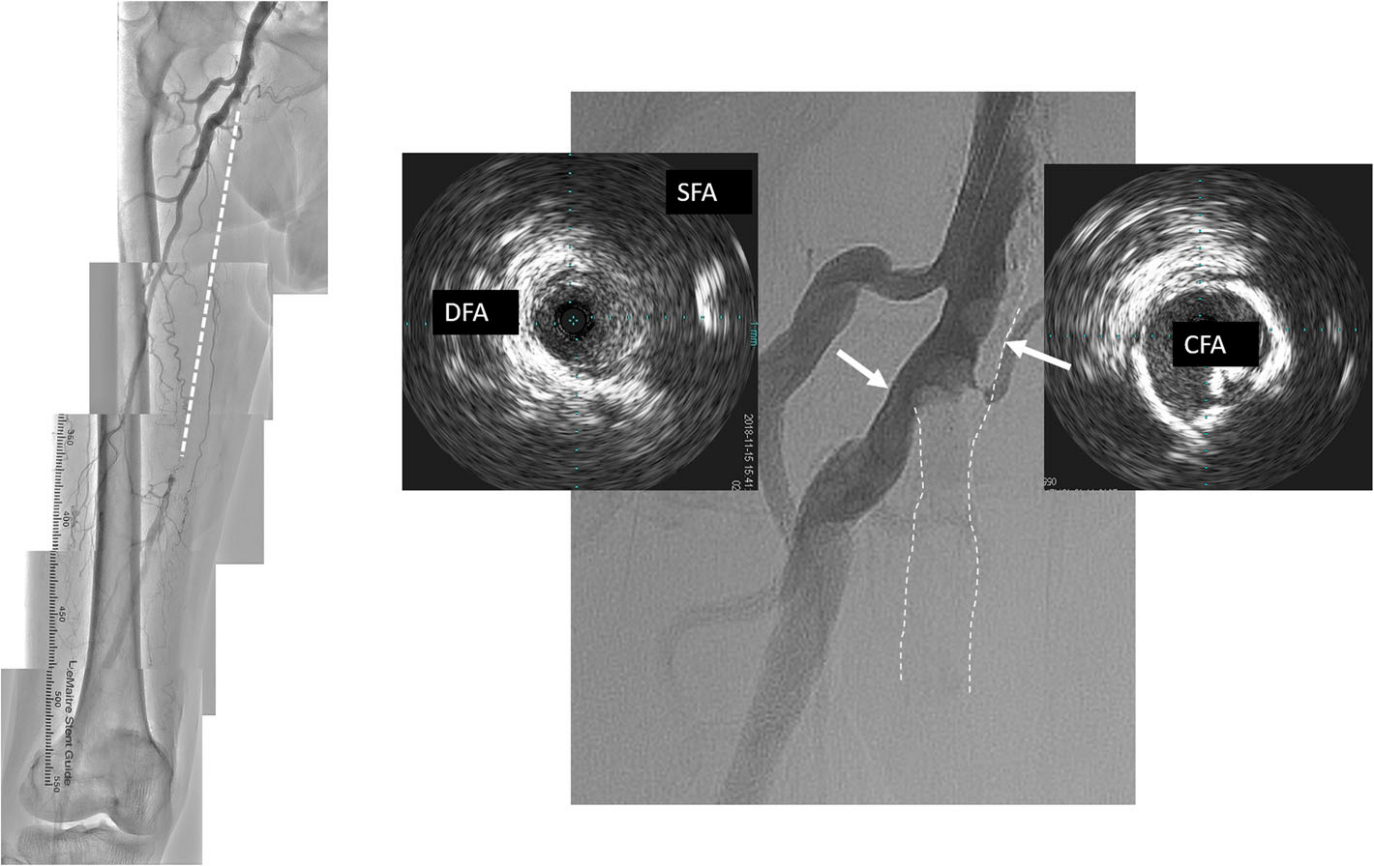
Angiography revealed a total occlusion in the right SFA. IVUS from the DFA revealed that the calcified plaque at the ostium of the SFA protruded into the CFA. SFA, superficial femoral artery; IVUS, Intravascular ultrasound; DFA, deep femoral artery; CFA, common femoral artery

A 78-year-old male, suffering from diabetes mellitus, presented with intermittent claudication (IC) of his right leg. He had a history of EVT for left SFA stenosis with severe calcification using Crosser® and a scoring balloon 3 years prior. One month prior, he became aware of the IC on his right leg and could walk only 100 m (Rutherford category 3). His right and left ankle-brachial indices (ABIs) were observed to be at 0.77 and 0.96, respectively. Both echography and angiography revealed a CTO proximal to the mid-SFA (Fig. 1). Before the invasive therapy, it was judged the calcification wasn’t so severe and the patient hoped to be treated with EVT. Therefore, elective EVT for this CTO lesion was planned. Conventionally, ASO patient with severely calcified lesions in common-superficial femoral artery recommend surgical treatment in our institution. However, EVT is selected alternatively in case the patient can’t be treated operation because the risk is high (e.g. advanced age, comorbidity, etc., ) or the patient wish strongly treatment of EVT not surgery.

The distance between the CFA puncture site and SFA ostium was at least 5 cm on the last contrast and it was judged that inserting the guiding sheath to the minimum treatable length was possible.

The right common femoral artery (CFA) was punctured using 18 G needle (Medikit Co, Tokyo, Japan) and good back flow of blood was confirmed. A 0.035-in guidewire (Radifocus®, Terumo Co., Tokyo, Japan) was inserted into the needle under fluoroscopy confirming no problem with wire movement and a 6-French guiding sheath (Parent Plus60 23 cm; Medikit, Tokyo, Japan) advanced about 5 cm via the ipsilateral.

Angiography revealed calcified plaque at the ostium of the SFA (Angiographic calcium score: Group4a [1]) (Fig. 1). Intravascular ultrasound (IVUS) (Vision PV; Philips, Amsterdam, Holland) was performed from the deep femoral artery (DFA) to confirm the lesion at the ostium of the SFA. It showed that the calcified plaque had protruded into the CFA (Fig. 1). It was supposed that stent implantation at the ostium of the SFA would lead to stent underexpansion, increasing the risk of the calcified plaque shifting to the orifice of the DFA. To avoid these complications, we have decided to remove the calcified plaque using myocardial biopsy forceps before inserting the guidewire to create a platform at the SFA orifice.

**Fig. 2:**
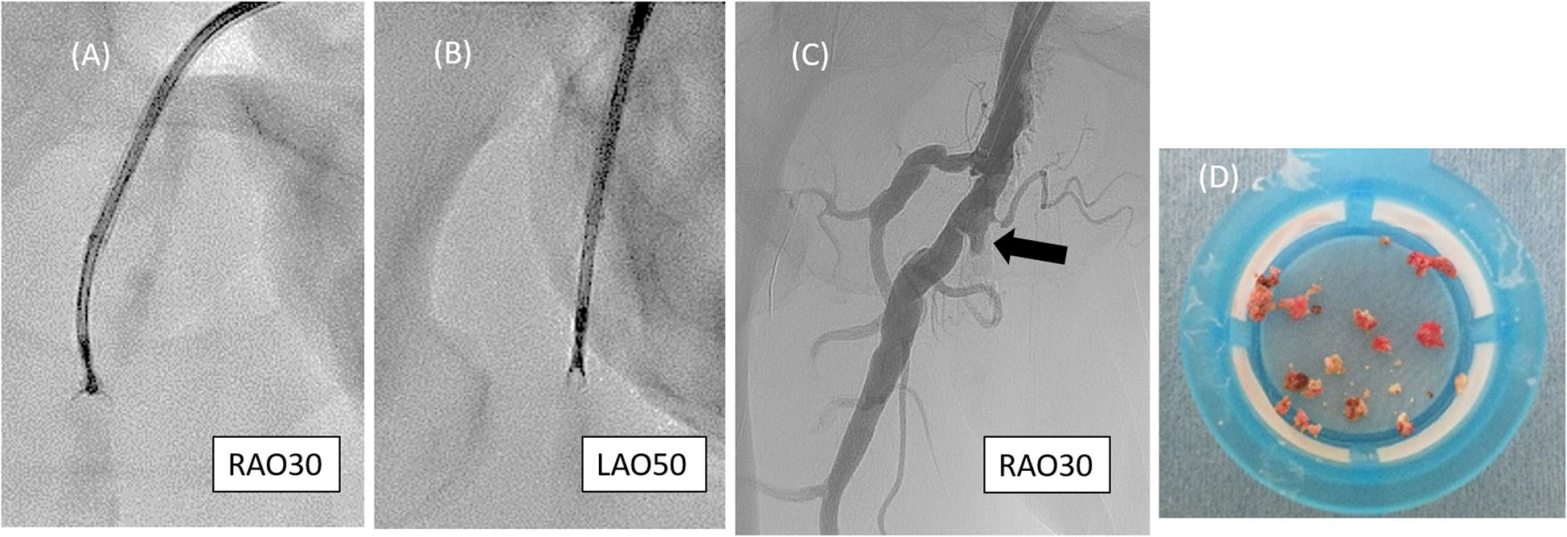
Removal of calcified plaque using the biopsy forceps. (**A**) The direction of the guiding sheath was adjusted by manually compressing the puncture site. (**B**) Contact between the open jaw of the forceps and the calcified plaque was checked using two perpendicular views before grasping. (**C**) The hollow at the ostium of the SFA (arrow) was created after repeating this procedure 21 times. (**D**) Removed calcified plaque. SFA, superficial femoral artery

**Fig. 3:**
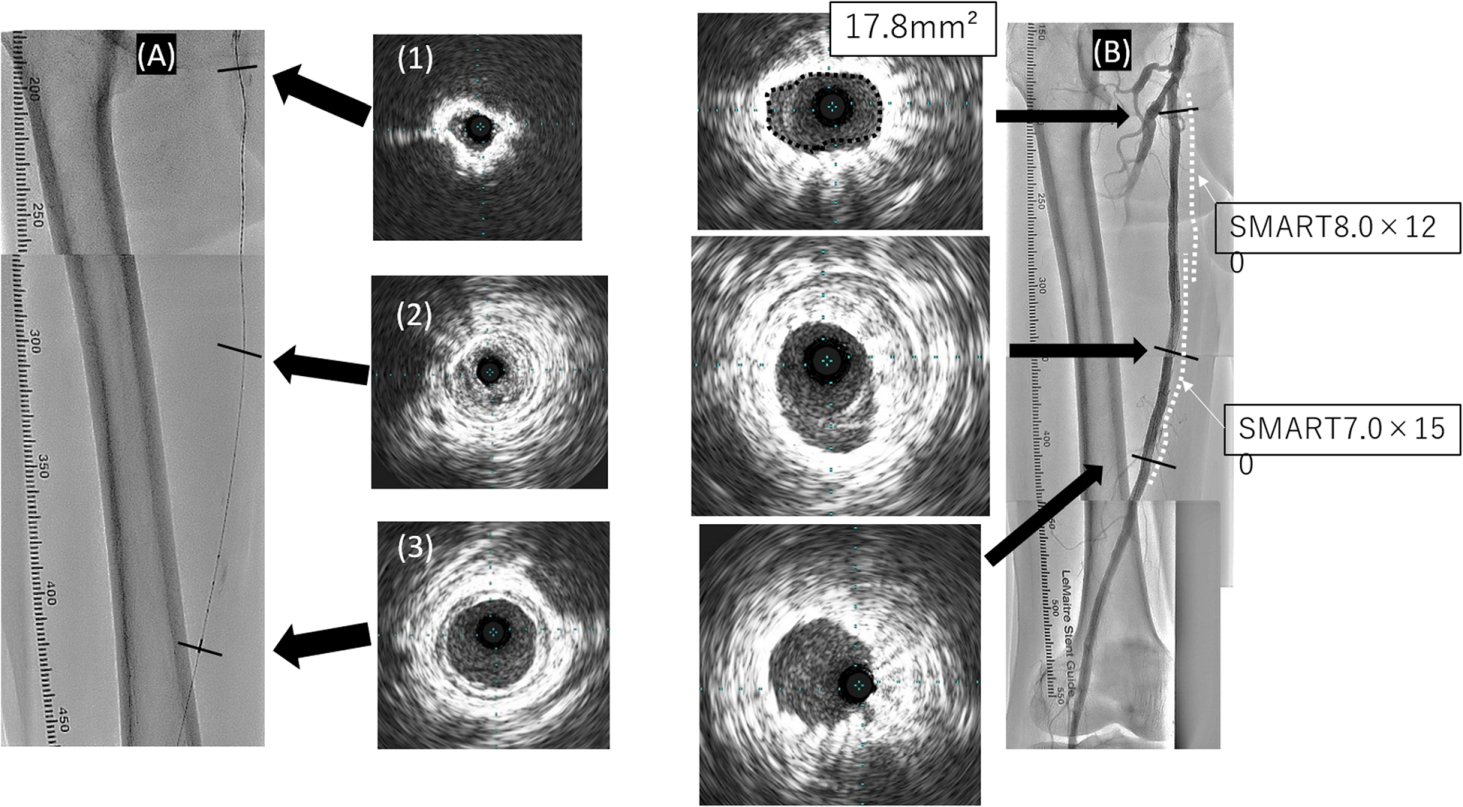
(**A**)IVUS images were obtained after the wire was passed through the CTO lesion. The vessel lumen at the ostium of the SFA was surrounded by calcification (1). The guidewire was passed through the center of the vessel (2). Distal reference of the CTO lesion (3). (**B**)Final angiography and IVUS. Acceptable expansion of the stent was achieved at the SFA ostium (17.8 mm²). Also, no plaque shift to the DFA was observed. IVUS, Intravascular ultrasound; CTO, chronic total occlusion; SFA, superficial femoral artery; DFA, deep femoral artery

The biopsy forceps (Technowood, Tokyo, Japan) were then inserted from the guiding sheath. To control the direction of the forceps toward the calcified plaque at the SFA, we compressed the puncture site using a finger to create a sheath tip facing the posterior wall to straight position toward the SFA. The biopsy forceps were opened, and the contact between the jaw and the calcified plaque was confirmed by two perpendicular views. After confirming the position, the jaw was then closed around the plaque, and the forceps were removed. These steps were repeated while fluoroscopy, and contrast injection was used to confirm the absence of dissection and/or perforation (Fig. 2). After 21 repetitions of this maneuver, a hollow at the ostium of the SFA was created. The jaw of biopsy forceps could not open due to the narrow space in the hollow. Therefore, we stopped these steps and begun inserting the guidewire. The endpoint of this maneuver was to create enough platform space at the ostial SFA. A 4-French tip angle 30° angle catheter (UNITE30; Asahi Intecc, Nagoya, Japan) was engaged at the hollow. A 0.014-in guidewire (VASSALLO40; Filmeck, Nagoya, Japan) was passed through the calcified plaque, successfully crossing through the distal part of the CTO using IVUS guidance. IVUS confirmed that the guidewire passed through the CTO lesion (Fig. 3).

After pre-dilation using a 4.0 × 20-mm scoring balloon (NSE PTA; Nipro, Osaka, Japan), two self-expandable stents (SMART 7.0 × 150mm and 8.0 × 120mm; Cordis, Florida, USA) were deployed from the SFA ostium. Final angiography showed TIMI III flow, and IVUS revealed an acceptable degree of stent expansion with a minimum luminal cross-sectional area of 17.8mm² through the entire lesion (Fig. 3).

After the procedure, the IC was completely alleviated, and the right ABI increased to 1.10. At the 9-month follow-up, the patient remained symptom-free with a right ABI of 1.06.

There are some caveats that need to be considered when using biopsy forceps to remove calcified lesions. First, calcified plaque must be clearly visualized using fluoroscopy. Second, the opened jaw of the biopsy forceps should be checked from multiple angles. Closing the jaw must be performed only when calcium is visible; otherwise, there is a risk of perforation. Third, the target lesion should form a linear anatomical line from the access site. This is necessary because the tortuosity of the vessel may prevent the opening of the biopsy forceps jaw. In general, when a crossover approach through iliac bifurcation is performed, the biopsy forceps cannot open. Fourth, the target lesion should have a vessel diameter just proximal to the calcified plaque that is just wide enough to control the direction of the forceps. The myocardial biopsy forceps (Technowood, Tokyo, Japan) that were used in this case were 5.7 mm wide at the jaw when fully open and the vessel diameter must be larger than 5.7mm when EVT treatment using this biopsy forceps. In small vessels, there is not enough space to open the jaw, and it is difficult to control the jaw direction. To control the direction of the biopsy forceps, it is efficient to make a gentle curve near the tip.

Embolic protection devices, such as Filtrap® (Nipro, Osaka, Japan) and Parachute® (Tri-med, Osaka, Japan), are generally recommended for use during this procedure due to the risk of fragment fail. In this case, we opted to not use these devices because the lesion was totally occluded and distal embolization only occur toward the DFA.

The endpoint of the procedure involves confirming the absence of the calcified plaque via fluoroscopy or determining that it is impossible to control the tip of the biopsy forceps.

Biopsy forceps may be used to reduce the total calcium volume in severely calcified vascular lesions. However, for the safety of the patient, the physician must be capable of performing this technique before attempting it.

## Conclusions

Biopsy forceps is one of the option for severely calcified lesion to reduce total calcium volume. It is most important to perform capable of ensuring safety.

## Data Availability

Not applicable.
